# The Role of Intuition in the Generation and Evaluation Stages of Creativity

**DOI:** 10.3389/fpsyg.2016.01420

**Published:** 2016-09-20

**Authors:** Judit Pétervári, Magda Osman, Joydeep Bhattacharya

**Affiliations:** ^1^Biological and Experimental Psychology, School of Biological and Chemical Sciences, Queen Mary University of LondonLondon, UK; ^2^Department of Psychology, Goldsmiths, University of LondonLondon, UK

**Keywords:** idea generation, evaluation, creativity, intuitive judgment, intuition

## Abstract

Both intuition and creativity are associated with knowledge creation, yet a clear link between them has not been adequately established. First, the available empirical evidence for an underlying relationship between intuition and creativity is sparse in nature. Further, this evidence is arguable as the concepts are diversely operationalized and the measures adopted are often not validated sufficiently. Combined, these issues make the findings from various studies examining the link between intuition and creativity difficult to replicate. Nevertheless, the role of intuition in creativity should not be neglected as it is often reported to be a core component of the idea generation process, which in conjunction with idea evaluation are crucial phases of creative cognition. We review the prior research findings in respect of idea generation and idea evaluation from the view that intuition can be construed as the gradual accumulation of cues to coherence. Thus, we summarize the literature on what role intuitive processes play in the main stages of the creative problem-solving process and outline a conceptual framework of the interaction between intuition and creativity. Finally, we discuss the main challenges of measuring intuition as well as possible directions for future research.

## Introduction

Celebrated mathematicians, scientists, painters alike often credit the role of intuition as part of the creative process that constitutes their discoveries (e.g., Hadamard, 1954; Gardner and Nemirovsky, 1991; Miller, 2000). For example, intuition was described as being at the core of creative visions of Steve Jobs, one of the foremost creative professionals in recent history (Isaacson, 2011). Yet despite this seemingly obvious connection between intuition and creativity, Dane and Pratt (2007), in their influential article, noted that “with the exception of a few studies (e.g., Raidl and Lubart, 2001), little empirical research has connected intuition to creativity” (p. 48–49), and this has been echoed by other researchers as well (Sinclair, 2010; Dörfler and Ackermann, 2012). In this article, we propose that though we cannot make a strong conclusion yet, there is, however, good conceptual grounds for proposing a link between the two, and promising evidence to suggest, that intuition and creativity are linked, at least on a minimal level.

The principal aim of the present review is to explore the potential link between intuition and creativity in a process-centric framework, in order to consider how intuition would be implicated in different phases of creative problem-solving. By intuition, we refer to its traditional characterization (Hogarth, 2001; Sadler-Smith, 2008; Dörfler and Ackermann, 2012), which treats the process as one which is rapid (also labeled as instantaneous), spontaneous (does not require extensive effort and cannot be voluntarily controlled), and alogical (does not necessarily follow the logical rules). Further, the outcomes generated from the intuitive process are generally holistic (also labeled as Gestalt as it is mainly concerned with the whole situation instead of its parts), tacit (the intuitive process cannot be verbalized or articulated with sufficient details), and made with high confidence. When a problem is complex, multidimensional and no pre-established clearly defined rules are available for solving it, a solution (i.e., a novel idea) is often based on the problem solver’s judgment of what is an appropriate solution in the absence of any clear, reasoned path. It is the contrast to developing a solution in a linear logically manner that makes idea generation characteristically intuitive and the idea itself that is opaque and inaccessible to the problem solver. Before establishing how intuition slots into different stages of the creative process, we first attempt to establish our conceptualization of creativity.

### Creative Problem-Solving Process

Creativity is a multifaceted construct and notoriously difficult to capture by a single definition (Runco and Jaeger, 2012). We conceptualize creativity as a process that is broadly similar to problem solving, in which, for both, information is coordinated toward reaching a specific goal (Wiggins and Bhattacharya, 2014), and the information is organized in a novel, unexpected way. For instance, Plucker et al. (2004) define creativity as “the interaction among aptitude, process, and environment by which an individual or group produces a perceptible product that is both novel and useful as defined within a social context” (p. 90). Problems which require creative solutions are ill-defined, primarily because there are multiple hypothetical solutions that would satisfy the goals (Reitman, 1965). Therefore, embarking on a solution to an ill-defined problem necessitates the problem solver to frame and interpret what might be relevant as a possible goal and then to establish a solution that meets that goal (Hayes, 1989; Mumford et al., 1994).

For a creative problem, an original solution is often unthinkable in advance, thus assessing creative solutions (i.e., creative ideas) occurs in the absence of objective criterion/criteria against which a creative product can be measured up to. As Amabile (1983, p. 359) put it, “current definitions of creativity are conceptual rather than operational; their conceptualizations have not been translated into actual assessment criteria” yet. Due to this “criterion problem,” it is difficult to objectively evaluate the extent in which a particular goal is met (Runco and Smith, 1992; Runco and Chand, 1995). Instead, various indirect features are used which often include, among others, the fluency, flexibility, originality, and elaboration of the solution (Torrance, 1966). It is questionable whether adding up the different features into a score of creativity does, in fact, constitute creativity, and whether, in fact, it should instead be the criteria by which the creative problem solver should assess a creative solution (Amabile, 1982).

The features by which a creative product is evaluated typically fall into categories that include novelty, feasibility, relevance, and specificity (Dean et al., 2006). It is here that intuitive judgments have been implicated with each of the categories related to evaluation. A creative problem solver may intuitively judge the creative product of the problem-solving process with regards to how novel the combination of information is, an intuitive recognition of the feasibility and appropriateness of the creative product, and the extent to which it seems like a good fit.

Turning now to the actual composition of the creative problem-solving process, there have been several ways in which this has been described. Most theorists assert that there are several consecutive stages (e.g., blind variation and selective retention model, Campbell, 1960; associative hierarchy theory, Mednick, 1962; three-process theory of creativity, Davidson and Sternberg, 1986; geneplore model, Finke et al., 1992). The number of stages differs by theory, and this is largely dependent on the ways in which theorists describe the critical components of the stages (e.g., preparation, incubation, illumination, and verification by Wallas, 1926; whereas problem formulation, preparation, idea generation, idea evaluation, and idea selection by Amabile, 1983). However, regardless of these variations, researchers agree on two main essential operations of the creative problems solving process: (1) the generation of ideas and (2) the evaluation and selection of (an) appropriate outcome(s) (e.g., Finke et al., 1992; Lubart, 2001; Reiter-Palmon and Illies, 2004).

Given that these two stages are common to all theories of creativity, and are relatively uncontroversial, it is for these reasons that this review focuses on these two stages as central to the creative problem-solving process. However, it is worth noting that the majority of the available literature tends only to investigate creative idea generation rather than idea evaluation (Amabile and Müller, 2009; Rietzschel et al., 2010). A further rationale for focusing exclusively on these two stages is that they can be explicitly related to how creative processes are measured empirically, and also help to conceptualize more easily where intuition as a process is directly associated with each of these stages, which we present in our framework in the concluding section of this review. Here we propose that both idea generation and evaluation are critical for shaping the creative product of the creative process, and that the two stages are tightly linked (neither makes sense without the other), and that the creative process is a dynamic one which can involve several iterations of generation and evaluation of ideas that a problem solver goes through before reaching an end state (Runco, 2003; Lonergan et al., 2004; Kozbelt and Durmysheva, 2007).

Regarding the underlying cognitive mechanisms, two antithetical types of thinking, convergent and divergent thinking (Guilford, 1956, 1967) are speculated to underlie both generation and evaluation of ideas in the creative problem-solving process. It has been proposed that problem solvers use convergent thinking for selecting a single (best) solution in response to a well-defined problem by applying standard procedures to existing knowledge. By contrast, divergent thinking can be utilized in more ambiguous situations, where a range of alternative solutions are possible, therefore responses may vary individually (Cropley, 2006). The popularity of the concept of divergent thinking has meant that for some it has been translated into a measurement tool of creativity itself (Zeng et al., 2011; Kaufman and Baer, 2012); though this approach has been severely criticized (e.g., Dietrich, 2007; Piffer, 2012). Among others, Cropley (2006) reset the balance by noting that both convergent and divergent thinking are necessary for producing creative ideas and that it is not simply contingent on divergent thinking alone.

Thus, to sum up, both idea generation and idea evaluation are two essential stages in creative problem solving, and in both stages, divergent and convergent thinking is utilized. Yet, no theory has provided the specific characteristics of intuition in these phases despite the speculation that intuitive judgment features throughout the creative process (Dane and Pratt, 2009). We propose here that intuitive judgment can be characterized in both idea generation and idea evaluation, and we spell out in our framework how this is the case.

### Intuition

Reaching a coherent perception of how to proceed toward solving an ill-defined problem is the key goal during both of the idea generation and the idea evaluation phases. Now we outline how intuition is defined and conceptualized related to this key goal. Bowers et al.’s (1990) classical model describes the process of intuition in two stages. In the first, guiding stage, clues (such as words, shapes, voices, odors, etc.) are accumulated from a complex, noisy environment and synthesized into a pattern in a gradual manner, resulting in a vague perception of coherence. If the spreading activation of relevant mnemonic networks exceeds a threshold, the perception of coherence becomes robust enough to enter awareness and results in a reportable hunch or judgment. This is interpreted as the second, integrative stage (see Volz and von Cramon, 2006; Zander et al., 2015 for neuroscientific evidence of this model).

We suggest that a perception of coherence underlies the finding of novel solutions. During the creative process, separate bits of information are acquired gradually. When embarking on a creative problem-solving process, the relevant prior representations/memories get activated from the accumulated prior experiences. These fragments are converted into a new unit that eventually reaches coherence. The novel organized whole (Gestalt) is assembled via associations, in a non-analytic and non-effortful manner. That is, a deliberate elaboration on how a novel product should be constructed would not count as intuitive.

Association-based information processing was found more appropriate than applying explicit algorithms or pre-established rules for solving complex problems by Dijksterhuis and colleagues (Dijksterhuis, 2004; Dijksterhuis and Nordgren, 2006; Dijksterhuis et al., 2006). Keeping in mind the task-specific goal but being distracted from it, coined as “unconscious thought,” was affiliated with association-based, bottom-up processing, as well as with a high processing capacity for solving multidimensional problems.

With regard to creativity, association-based processing serves as a good foundation for generating original responses. As noted by Gallate and Keen (2011), using intuition means not pursuing “a consciously deductive path and is, therefore, more likely to be original because it does not build on something that is already ‘known”’ (p. 686). Essentially, taking the claims here as a point of departure, big leaps often found in the creative process might be thought to happen if creative problem solvers are not fixed on the rules of a current paradigm (e.g., set out to optimize aspects of an already existing structure), rather, this will happen when solutions are generated independently, keeping in mind the desired end state and making individual judgments on how to get there instead of relying on what has been put forward already. Individually tailored responses are more diverse and more likely to converge toward a unique outcome than those building upon existing structures.

Many times, individual, association-based responses must be formed to complete a task-specific goal. Intuitive processes are even categorized based on the domains to which these goals are connected: (1) problem-solving, (2) creativity, and (3) moral judgments (Dane and Pratt, 2009), as well as (4) social judgments (Gore and Sadler-Smith, 2011). As an alternative typology, Glöckner and Witteman (2010) unpack the sub-categories of intuition based on its underlying cognitive mechanisms, i.e., they lay out associative intuition, matching intuition, accumulative intuition and constructive intuition as partly overlapping but differently focused intuitive processes. Glöckner and Witteman’s (2010) approach is distinct from the domain-based approach yet still consistent with it, e.g., matching intuition can be easily related to problem-solving intuition, or constructive intuition appears to form part of creative intuition. We consider creative intuition as key to idea generation, and problem-solving intuition as key to idea evaluation.

### The Link between Intuition and Creativity

Although various researchers have reported a close connection between intuition and creativity (e.g., Perkins, 1992; Boden, 1994; Policastro, 1999), a precise spelling out of how these two constructs are linked has not yet been adequately established. In the main the reason for this is largely the result of the common observation that there is only scarce direct evidence at hand on the particular role of intuition in the creative problem-solving process (e.g., Agor, 1989; Policastro, 1995; Shirley and Langan-Fox, 1996; Dane and Pratt, 2009; Eubanks et al., 2010; Sinclair, 2010; Stierand and Dörfler, 2015), and due to a lack of such evidence, more empirical work is needed (e.g., Raidl and Lubart, 2001; Dollinger et al., 2004; Dane and Pratt, 2007).

As we have proposed earlier, idea generation and evaluation are stages of creative problem solving. They are both found in unstructured and ill-defined problems that have no pre-defined objective criterion to measure against to the product of the creative process. As mentioned in the previous section, the complication is that stating explicit rules is unworkable when it comes to creating novel and/or original solutions, also because often there are no objective rules. Thus we propose that intuitive judgment is an important feature in the creative process, for this reason that people often lack insight into how they generated a novel solution, and experience surprise, i.e., the violation of previous expectations related to the solution is phenomenologically often at the heart of perceiving something as creative (cf. *effective surprise*, Bruner, 1962; Wiggins and Bhattacharya, 2014). Because there are no objective rules on how to reach a solution to a creative problem, a combinatorial explosion of possible choices occurs (Simon, 1989; Simonton, 2010). Relying on intuition is a common tool for coping with such a complex and noisy environment, somatic signals are often guiding the early stages of the creative process (Finke et al., 1992; Hodgkinson et al., 2008).

During the integration of information both while looking for novel patterns (idea generation phase) and while assessing them against prior experiences (idea evaluation phase), an internal sensing of which choice alternatives have the most potential can direct attention away from selecting predictable solutions. A creator proposing ideas which rely heavily on previously acquired information is more likely to generate solutions that are predictable, as compared to a creator relying on hunches about unknown, new directions which would more likely lead to surprising solutions (Simonton, 2012). These hunches cannot be well described with words (Sadler-Smith and Shefy, 2004) and are largely different from having a sudden stroke of insight (e.g., Hogarth, 2001; Dane and Pratt, 2007). As insight is often considered a hallmark of creative problem solving, and there are common practices of using these two words in an interchangeable fashion, we note that there are considerable differences between these concepts. In contrast to the aforementioned characteristics of intuition, we propose that gaining an insight means that the problem solver obtains an explicit understanding of how to reach the goal (Lieberman, 2000), and is capable of articulating it too (Dane and Pratt, 2007). While intuitions unfold gradually, “Aha!” moments are experienced in a discontinuous manner (Zander et al., 2015), as if a light bulb is switched on in the problem solver’s head (Jung-Beeman et al., 2004; Slepian et al., 2010).

In contrast to the definitiveness of an insight, intuitions are more indefinite. E.g., creative intuition is described as “a vague anticipatory perception that orients creative work in a promising direction” (Policastro, 1995, p. 99). What’s more, it has been conceptualized as “a tacit form of knowledge that broadly constrains the creative search by setting its preliminary scope” (p. 100) as well as a guide for discovering new ideas and assessing whether the idea is appropriate for a problem (Dollinger et al., 2004). However, creative intuition utilized at the early stages of the creative process seems to be only one side of the coin (Policastro, 1995; Raidl and Lubart, 2001; Dane and Pratt, 2009).

We suggest that not only creative intuition but other types of intuition too are relevant for creativity. Namely, we propose that problem-solving intuition (Dane and Pratt, 2009; Gore and Sadler-Smith, 2011) is employed during the later stages of the creative process. This type of intuition is defined as a “domain-specific, expertise-based response to a tightly-structured problem based on the non-conscious processing of information, activated automatically, eliciting matching of complex patterns of multiple cues against previously acquired prototypes and scripts held in long-term memory” (Gore and Sadler-Smith, 2011, p. 307).

If we compare the two functions on which our conceptualization of intuition emerges, they can be seemingly contradictory. The contrast being that creative intuition employed during the idea generation phase relies chiefly on synthesis, while problem-solving intuition operating during the evaluation phase is frequently tied to analysis. That is, in the idea generation phase, creative intuition can work as an associative process linking together distinct pieces of stored information and restructure/combine them into a coherent, task-relevant unit. Akin to constructive intuition (Glöckner and Witteman, 2010), mental representations are constructed based on both current information and traces activated from long-term memory.

In the idea evaluation phase, expertise related to the recognition of novel contributions and judgment regarding whether the product would be perceived as appropriate in a given social context must be drawn upon. Usually, this operation is performed by matching stimuli to already acquired prototypes, however, creative solutions may be special in that they are likely to alter from previous prototypes. In extreme cases, a surprising creation might not fit any existing prototypes, which can also make it difficult to assess its significance in the context in which it was generated. If an idea is unlike the judge’s earlier experience, clues to its coherence must be evaluated.

### Reviewing the Evidence on the Link between Intuition and Creativity

Before we go on to lay out our proposed framework, we now consider of the extant empirical findings regarding explorations of the link between intuition and creativity. The empirical findings are presented according to the type of research (qualitative/quantitative) and phase of the creative problem-solving process (idea generation/evaluation) they explore. What follows after the review is a summary of the main difficulties of measurement and assessment of the association between intuition and creativity, and a recommendation of a way forward based on our new conceptual framework, and possible future research directions that logically follow from it.

## Methods

### Literature Search

We first performed an extensive search of relevant databases, namely used the Web of Science, PubMed, PsycINFO, Google Scholar, and Scopus. The search was conducted using the following keywords: creative, creativity, creative evaluation, insight, innovation, divergent thinking with the Boolean operator AND linking intuition, intuitive problem solving, and decision-making to them. Through the use of these broader terms, we, therefore, incorporate studies focused on more specific ideas within these terms, such as the idea generation and idea evaluation expressions. Though we have not specifically used idea evaluation, in wider literature, this term is used interchangeably with one of our selected keywords, creative evaluation.

For selecting keywords, we started at baseline terms: creativity and intuition. After conducting a literature search with these, we chose to include additional terms which were both common and could possibly incorporate further relevant studies in our search. Additionally, theses and dissertations were retrieved from the British Library EThOS and from the Open Access Theses and Dissertations databases. The citations of studies were examined in order to obtain further relevant empirical work regarding the link of intuition and creativity.

### Inclusion Criteria

Two criteria were applied for inclusion of studies: the research must be (1) empirical work and (2) taking both intuition and creativity into account. Thus research investigating only intuition *or* only creativity was not included in this review. Results were filtered from sole phenomenological descriptions and work diaries lacking any qualitative or quantitative analysis, as well as from parapsychological investigations since they did not fit the scope of the article. Individual testimonies, historical studies, and biographies (e.g., Policastro, 1995) were also not included here. Further, creative performance must have been demonstrated either by professional track record or by completing creative problem-solving tests, studies relying solely on self-report questionnaires to determine creative potential were not considered here. These procedures yielded a pool of 70 potential studies from which 11 fulfilled all of the aforementioned criteria. **Table 1** includes the list of papers organized by the timeline of the creative process.

**Table 1:** Matrix of the analyzed work.

Research	Source	Type of research	Concept used for intuition
On intuition and creative idea generation	Marton et al. (1994)	Qualitative study	Scientific intuition
	Garfield et al. (2001)	Experimental study	Intuitive cognitive style
	Raidl and Lubart (2001)	Correlational study	Intuition (captured by multiple measures)
	Eubanks et al. (2010)	Experimental study	Intuition (correct, rapid, self-reliant)
	Sinclair (2012)	Qualitative interview	Intuitive expertise, intuitive creation
	Dörfler and Eden (2014)	Qualitative interview	Intuition, big leap
	Stierand and Dörfler (2015)	Qualitative interview	Intuitive insight
On intuition and creative idea evaluation	Sinclair (2012)	Qualitative interview	Intuitive foresight
	Magnusson et al. (2014)	Experimental study	Intuitive assessment
	Eling et al. (2015)	Experimental study	Intuitive analysis/decision-making
	Stierand and Dörfler (2015)	Qualitative interview	Intuitive judgment
On intuition and creativity (no differentiation between the stages)	Dollinger et al. (2004)	Correlational study	Intuition
	Sundgren and Styhre (2004)	Qualitative interview	Intuition (Bergson’s definition)

## Findings

Studies found within our literature review will be presented below according to their relation the main stages of creativity, i.e., idea generation or idea evaluation.

### Studies on Intuition and Creative Idea Generation

Experts of different domains have been interviewed in order to gain insight into the role of intuition in their idea generation process. Dörfler and Eden (2014) reported the common patterns emerging from face-to-face interviews with 17 Nobel laureates and two Eckert–Mauchly prize winners. Marton et al. (1994) analyzed answers to short, prearranged interview questions across a larger sample from footage of a television program “Science and Man” across 14 years, totaling 93 Nobel Laureates from physics, chemistry, and medicine. Marton et al. (1994) grouped the reported experiences according to (1) when intuition was defined as an outcome, (2) as an act or event, or (3) as a capability. Seventy-two of the 93 respondents expressed a belief that scientific intuition does exist, and from those 28 saw it as a capability, 20 as an act or event, and eight as an outcome, and even these last respondents suggested that it formed part of the starting stage of the creative process. Apart from describing the frequencies of the responses given by the Nobel laureates, Marton et al.’s (1994) study only reflected the scientists’ naïve understanding of the issue and was inconclusive about the interpretation of the results with regards to a precise link between intuition and creativity.

In contrast, Dörfler and Eden (2014) analyzed the transcripts of lengthy interviews conducted with a smaller sample (*n* = 19). They identified three common themes: (1) the role of a “big leap” and how intuition contributes to big scientific discoveries, (2) the significance of having a dual-view, i.e., processing information both globally and a locally (Dijkstra et al., 2012; Förster, 2012) and (3) what is a common structure of successful research teams. All of the respondents confirmed they utilize their intuition during the scientific inquiry, even if they avoided using the exact term due to its pejorative connotation. Instead, Dörfler and Eden (2014) treated references to big leaps as situations showing evidence of intuition, “where a step in thinking is made that does not logically follow from a process of analysis; rather the process of analysis follows the big leap and is used to justify the ‘big leap”’ (p. 5).

There has been some work examining professions connected to artistic creativity, namely the creation of haute cuisine served by fine dining restaurants, and filmmaking. While the aim of Stierand and Dörfler’s (2015) study was to find out more about the creative process of turning raw ingredients into delicious dishes, the theme of intuition emerged from their interviews. The in-depth reports from renowned European chefs revealed that they rely on intuition both during the generation and the screening of ideas. The self-reported experiences were classified as either (1) intuitive insight or (2) intuitive judgment (Dörfler and Ackermann, 2012). Intuitive insight was conceptualized as a resource during which chefs’ mentally combined ingredients and developed a gut feeling about which combination should be tested. The researchers identified the role of intuition as a rapid coupling between the idea generation and the idea evaluation phases providing feedback loops for the iterative creation process.

In regard to film production, Sinclair (2012) interviewed 47 filmmakers between the age of 26 and 71 and with 8–42 years of domain-related experience classified their job as primarily creative (11 directors, three architects, three screenwriters, six directors of photography), primarily technical/operational (12 production managers) or primarily strategic (nine executive producers, two studio directors). The responses recorded in the interviews were clustered into three main categories: (1) intuitive expertise, (2) intuitive creation, and (3) intuitive foresight. The extent in which filmmaking professionals utilized intuition differed according to job specialization. Intuitive creation was demonstrated only by creative film professionals when they approached the story or visualized the set, conceived characters/shots, created (visual) storylines, or gave instructions to actors. Taken together, qualitative studies revealed personal insights regarding the experiences of intuition in the creative process amongst professionals across a variety of sectors. In the main, the common insights appear to be interviewees spontaneously report that intuition is an essential part of the creative process. Moreover, they rely on their intuitive capacity to find new directions of inquiry leading to discoveries they would not have otherwise have made, as well as judging the success of their creative solutions.

Compared to the limitations of using qualitative methods, quantitative study designs can capture a larger, but non-expert, sample. In practice, the most common approach has been to use psychometric assessments to capture individual differences in the intuitive processing in creativity through questionnaires. Intuition and creativity are heterogeneous concepts, and particular components of them are likely to be correlated in various ways; Raidl and Lubart’s (2001) study involved several measures. As a measure of creativity, they used Torrance’s Unusual Uses Test (Torrance, 1966). This involved participants generating as many and rare uses as possible for a cardboard box. Amabile’s (1982) Consensual Assessment Technique was used to assess two further creative production tasks which involved participants producing a drawing from a set of graphical elements, and creating a short story from just a title.

On the other hand, intuition was assessed using the Rational-Experiential Inventory (REI, Epstein et al., 1996), in which preferences for rational versus experiential information processing were scored based on Likert-scale, and the Intuitive Behavior Questionnaire (IBQ) in which participants faced a problem and selected a solution that could be either an intuitive or an analytic one. In addition, two behavioral measures of intuition were also presented. In one of them, participants had to group 8 abstract images in multiple ways, giving a title to each grouping. The responses were analyzed by judges who classified the groupings either as intuitive or analytical. The other involved presenting participants with 10 items were taken from the Metaphoric Triads Task (Kogan et al., 1980), each item corresponding to three words or three images which could be associated either via a metaphorical or a functional link. Preference for the metaphorical and not the physical link was counted as an intuitive response.

The results from this battery of tests presented to 76 undergraduate psychology students revealed that IBQ scores correlated with drawing production, and with the fluency and mean originality scores on the Unusual Uses Test. The high intuition group, assessed by the IBQ, scored higher on the creativity measures than the low IBQ group. REI test performance correlated positively with the drawing production task performance, the metaphor preference test performance, and the mean originality score on the Unusual Uses Test.

In a further study by Garfield et al. (2001), intuition was measured by the most commonly used measure of intuition, the Myers–Briggs Type Indicator (MBTI). MBTI makes use of binary distinctions of personality types based on the scores of its extraversion–introversion, sensing–intuition, thinking–feeling, and judging–perceiving subscales (Myers and McCaulley, 1985). The MBTI takes Jung’s idea that personality types are connected to conscious and unconscious working methods of the mind (1921/1971), and has adapted it to assess dimensions of personality, of which the “intuitive type” is one. Myers and McCaulley (1985) conceptualized intuitive types as those that form perceptions which are oriented to the future and concerned with seeing previously undetected patterns.

Garfield et al. (2001) used the MBTI with participants who were trained either an analytical or an intuitive problem-solving technique (VanGundy, 1988; Couger, 1995). Creativity was measured by the Kirton score (Carne and Kirton, 1982), which categorizes problem solvers as either adaptors or innovators and expects them to come up with either paradigm-modifying or paradigm-preserving ideas accordingly, and was manipulated by presenting the participating 219 undergraduate business students with novel or not novel ideas “from others.” The group which used the intuitive problem-solving technique came up with more novel and paradigm-modifying ideas as contrasted to those who used the analytical technique. Also, participants exposed to novel and paradigm-modifying ideas from “others” generated more novel and paradigm-modifying ideas themselves, and vice versa.

The influence of intuition on idea generation process was also examined by Eubanks et al. (2010). This research aimed to show direct evidence for the link between intuition and creative problem-solving by manipulating affect and level of training, both treated as facilitators for using intuition. Participants’ affect was manipulated at the beginning of the experiment by playing music that was designed to induce positive affect in one group, and a neutral experience in the other group. All participants, except the control group, were then trained through instructional exercises to use their intuition to solve a series of creative problems Participants were classified as being intuitive if they were above the group average in providing correct answers, below the average in solution time and below the average in utilizing optional additional information for the problems. Training made a strong positive contribution to creative problem-solving performance (measured according to the quality, originality, and elegance of solutions to the problems) in general. When a neutral affect was induced, intuition scores were strongly associated with enhanced creative problem-solving performance. When positive affect was induced, the association between intuition and problem-solving performance was undermined, and it alone did not lead to any creative performance advantage alone and in the control group which received no instructional training.

These studies have been grouped on the basis that they employed questionnaires to quantify the intuitive and creative abilities of students. All demonstrated a positive association between generating new ideas and relying on intuitive resources, including the production of more novel, higher quality, and more diverse ideas.

### Studies on Intuition and Creative Idea Evaluation

Idea evaluation is a more scarcely used term within the literature, with a few studies combining this concept with idea generation, and even fewer assessing this concept in isolation. We have introduced two studies (Sinclair, 2012; Stierand and Dörfler, 2015) in the previous section which predominantly discuss the concept of idea generation but also include short passages on idea evaluation. Both studies introduce new terminology to describe similar concepts with functional differences. We coordinate these with our framework.

Stierand and Dörfler (2015) introduced intuitive insight and intuitive judgment as mechanisms underlying creative discoveries. From these, intuitive judgment may be applied in the creative evaluation stage, e.g., deciding the array of dishes on a menu. An additional term introduced by Sinclair (2012), intuitive foresight, can also be connected to idea evaluation. According to her data, both intuitive expertise and intuitive foresight were used by all filmmaking professionals. Intuitive expertise functioned as a way to create unity amongst crew members whereas intuitive foresight was crucial for making decisions regarding the selection projects, topics/script, and for helping spot talent or market trends.

Two studies we examined focused exclusively on the idea evaluation stage. In the first one (Magnusson et al., 2014), expert judges carried out the evaluations of products. Intuitive idea evaluation was compared with analytical idea evaluation against predefined criteria in the context of developing new products. Clients of a big telecommunications operator were asked to submit their ideas on developing future mobile services. Eighty-three separate ideas were evaluated by four experts—one of whom also provided qualitative data as part of a thinking-out-loud protocol but due to the limited sample size this data is not reported here. All four judges evaluated each idea first in a holistic manner (intuitively), and then 2 weeks later according to formal criteria (analytically). Intuitive evaluations were made while keeping first a radical and then an incremental market in mind, while analytical evaluations were made according to three formal criteria, namely originality, user value, and producibility. A link between the two techniques was shown with linear regression. The analysis showed that the scores on the three formal criteria predicted approximately 50% of the variance in the holistic evaluations. Furthermore, two innovation indexes (based on Magnusson, 2009) were calculated, with which the best ideas from both the incremental and radical perspectives were selected.

In a similar vein, Eling et al. (2015) also investigated the role of intuitive and analytical evaluation processes during early idea screening by utilizing Dijksterhuis’ (2004) research design. Fifty professionals that were qualified in product development were presented with four new product ideas, each consisting of 12 attributes. After briefly reading one of new product ideas participants could either perform a rational analysis (i.e., deliberately assess the idea in a logical manner) or complete a distractor task for the equivalent length of time (i.e., 3 min) after which they were required to rely on their “intuition and gut feeling” about the new product idea. Another group was exposed to both, in the order of rational analysis then intuition (via the distractor task), and a final group was exposed to the intuitive then rational analysis. The combined approach of intuition and rational analysis increased the speed and quality of the evaluation of the new product ideas rather than rational analysis or intuition alone, the latter of which would have been predicted by Dijksterhuis (2004).

In conclusion, larger creative outcomes can only be examined by breaking them down into smaller building blocks and tracking how they influence the final product. These studies followed real-life examples of creative achievement from beginning to end, interpreting evaluation through the attrition of lower quality ideas within each building block. In addition, it was shown that there is more to intuitive evaluation than a rapid use of criteria since an analytical evaluation could explain only half of the variance shown in the intuitive assessment. Combining intuitive and analytical approaches led to higher quality and faster idea evaluation than relying on one of the approaches only. Considering the low number of studies conducted on idea evaluation, further research efforts would be necessary to explore the exact role of intuition within this stage.

### Studies on Intuition and Creativity (with no Differentiation between the Stages)

Two of the found studies did not decompose the creative process into multiple stages, but made general claims and focused on the details of intuitive processes. Sundgren and Styhre (2004) focused their work on scientific research and narrowed their scope to a case study of pharmaceutical research. Particularly, the organization of pre-clinical drug development, employee’s understanding of the concept of intuition, intuition’s role in the discovery of new drugs, as well as moderating organizational factors were recorded. The narrative analysis of the interviews resulted in a list of characteristic experiences, however, the contents were not quantified nor fit into a larger context. Nevertheless, the key quotes served as valuable sources for enhancing insider understanding and inspiring further research.

Dollinger et al. (2004) used the MBTI along with several other creative performance measurements to explore the link between intuition and creativity. In their study, 94 college students completed a shortened version of the Creative Behavior Inventory (Hocevar, 1979; Dollinger, 2003), the Creative Personality Scale (Gough, 1979) and produced a drawing as part of the Test for Creative Thinking–Drawing Production (Urban, 1991; Urban and Jellen, 1996). Consistently with past research (Myers, 1998), participants who were classified as both intuitive and feeling types scored the highest on the creativity tests, while the lowest scores were associated with those identified on the MBTI as Sensor-Feeler types. Though these studies reinforced general notions about intuition contributing to discoveries/creative productions, they were unable to outline new directions for further expansion.

## Discussion

The aim of the present review was to examine the link between creativity and intuition with a special emphasis on how intuition fits into the specific stages of creative processes. We decomposed creativity into idea generation and idea evaluation phases and considered two types of intuition, creative intuition, and problem-solving intuition. Creative intuition was linked to the idea generation phase, whereas problem-solving intuition was linked to the idea evaluation phase. It was hypothesized that a gradual accumulation of clues to coherence underlies the generation and recognition of creative ideas, as reaching a coherent perception of how to proceed is the key goal during both of the idea generation and the idea evaluation phases in the absence of consensually accepted rules.

We categorized available research literature into three sections based on the proposed conceptual framework. The majority of our findings were concerned with idea generation, which could reflect the common belief that creativity arises from idea generation. Qualitative studies suggested that intuition was relevant for creativity but this was based on introspection and anecdotal evidence, albeit given by professionals in their own respective fields. What we could infer here is that these two constructs are likely to be connected but it is not known how they are connected. Correlational studies showed a reasonable correlation between intuition and creativity, but there may well be conflation given that the creativity and intuition measuring instruments may include similar items. Finally, empirical studies showed that intuition may guide idea generation and evaluation, and optimal performance was achieved when analytical and intuitive judgments were combined.

Taking these findings into consideration, we can conclude that the exact ways through which intuition is connected to the different stages of the creative process still need to be empirically demonstrated. However, they do suggest that for ill-defined problem scenarios where the number of possible solutions increases to near-infinity, creative thought starts with intuition and intuition is inherently part of the process. In order to examine this connection, there need to be a clear set of hypotheses to test regarding the precise nature of the relationship. We propose a framework that makes this possible which is also informed by the current evidence reviewed, We draw attention to the fact that thus far, no existing theories of creativity have included intuition as a component prior to our framework. Our aim is to lay out a framework which establishes the timing and magnitude of the contributing intuitive process make to the creative process. But, before we present the framework, we discuss a few limitations.

### Limitations

To begin, the review represents specific literature that may be construed as biased in the following ways. We only considered the period after the first landmark review of the psychological evidence connecting creativity and intuition (Policastro, 1995). Further, our selection criteria were strict which in turn mean that this only generated a handful of studies that could be included in the review. Furthermore, this review does not represent the entire spectrum of studies relevant to the main topic, because of the stringent exclusion criteria which did not include main streams of research (e.g., excluding the studies featuring self-reports only). We wanted to keep a sharp focus on the most directly relevant evidence available on the topic of the connection between intuition and creativity, with the view to only including high-quality literature that provided insights that directly concerned the connection between the two phenomena of interest. Thus while we have indeed used self-imposed filters in this review but these filters we presented a clear justification for them earlier in the Section “Methods” of this article. The goal was to gain a deeper understanding of the connection between the two concepts and to be able to start moving forward with the experimental work from there.

One concern regarding using the reviewed literature to potentially inform our framework is the difficultly in synthesizing it. Questions can be raised about what we can take away from the findings discussed from the literature given the different conceptualizations and operationalizations about the core phenomena being investigated. In addition, a further related problem concerns the misaligned assumptions surrounding both intuition and creativity and the way in which they are measured. Another issue concerns the topic of examining the connection between intuition and creativity itself, which confronts the edges of our current discipline’s understanding of the operations of knowledge integration at a cognitive and neural level (Park and Friston, 2013).

Thus, for now our review, while broadly informed by the empirical literature, does not have a dedicated set of studies to support it. However, the aim here is to find common ground in theoretical and empirical work, in order to provide testable hypotheses about the linkage of the processes couched in a detailed conceptual framework.

### Conceptual Framework of the Link between Intuition and Creativity

**FIGURE 1:**
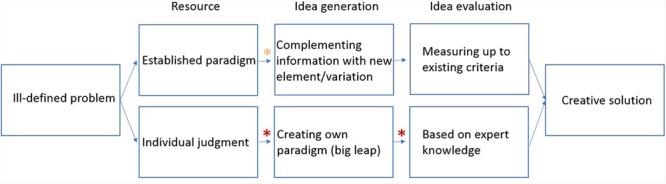
**Conceptual framework of the place of intuition in the creative problem-solving process.** The red asterisk denotes stages at which intuition is necessary to proceed to the next phase, whereas the orange asterisk highlights a stage at which intuition might be applied to proceed to the next phase. Note that most problems can be solved via both pathways.

Our aim here is to present a framework that is able to consolidate the essential features of the creative problem-solving process, and intuition (more specifically intuitive judgment), and to lay out how the two are connected. Moreover, the aim is to show sensitivity to the insights from theoretical and empirical work that has speculated a link between intuition and creativity. In order to follow our proposals, **Figure 1** presents a schematic of our conceptual framework, and the elaboration of the framework that follows discusses the components from left to right as they appear in **Figure 1**.

Ill-defined problems are the starting point of the creative problem-solving process, and once a creator faces such a problem, they can begin tackling it in one of two possible ways: (1) they may refer to existing prescribed paradigms (these may be institutional depending on the context in which the problem arises) to define the problem space and the possible strategies that could be taken, or (2) they may use one’s individual judgment based on prior experiences to define the problem’s characteristics.

#### Path 1

Selecting an established work frame to tackle a problem may seem initially efficient, but may also be unsuitable for reaching the goal, thus ultimately lead to an insufficient solution or no solution at all. However, the advantage, along with efficiency, is that later down the process of creative problem solving, solutions/innovations may be achieved by committing to established paradigms and inserting new elements into the framework or finding a beneficial variation of existing elements based on accumulated cues to solve a problem. The underlying assumption is that the existing framework is sufficient for reaching the goal (in many cases it is the optimization of the process by which the goal was achieved already), thus it is used as a starting template to build upon.

Within an already established framework, it is relatively easier to assess the potential and actual value of new propositions. These newly proposed alternatives are comparable with the prior less elegant/optimal solutions and often there is a general set of criteria for judging their value. During this first pathway, intuition may be employed to recognize new elements or variation of elements by recognizing their value based on gut feeling. However, rational analysis may yield the same results through a less elegant, more time-consuming procedure. It is thus Path 2 in which intuition is more obviously featured in both idea generation and evaluation.

#### Path 2

In contrast, big leaps in knowledge occur if problem solvers create a novel paradigm to solve a problem and this can serve as the basis for solving future, related problems. The motivation for doing so is that the existing framework proved to be unproductive for reaching a specific goal, such as there may be empirical evidence at hand which does not fit the theoretical assumptions, or a problem must be solved which cannot be asked/answered under the existing frame. It is also possible that a creator is not knowledgeable of existing procedures thus establishes their own. Deliberate analysis is ruled out here because a thorough evaluation of a vast amount of randomly generated possibilities would not be feasible due to a lack of resources (time, funding, etc.). The same applies to relying on chance and selecting ideas completely randomly. Rather what happens is that a creator gains a starting hypothesis relying on a gut feeling. He/she combines separate chunks of gradually acquired information about what could be working and boils them down to form a new coherent construct via associations. Intuition does not solve the entire problem but grants an idea which is purposefully selected. In this path, intuition cannot be replaced with analysis and it sometimes even precedes analysis (Dörfler and Eden, 2014). It is tightly linked to establishing new paradigms, not only in the idea generation phase but in the evaluation phase too. Initial ideas need refinement and must be monitored based on how close is the current state to the desired end state. Experts of a particular domain must rely on their perception of coherence to judge the explanatory potential of a new framework (whether it is suitable for addressing the question and what further problems may get answered with it).

### Directions for Future Research

Further experimental studies are necessary to investigate the proposal here. In particular, based on the predictions made, future investigations should explore whether well-defined problems involve intuitive solutions. In addition, it would be useful to test whether a truly creative paradigm, which incorporates three essential criteria, i.e., originality, utility, and surprise (e.g., Simonton, 2012), can be generated by relying solely on analytical methods. Furthermore, to answer the question whether intuition is indispensable for creative achievements, scenarios in which only intuitive processing of the problem, only analytical processing of the problem and both intuitive and analytical processing of the problem is carried out should be contrasted (cf. Eling et al., 2015). Studies usually contrast intuitive judgment to analytical judgment, so it could be worthwhile to look specifically at association- versus rule-based judgments during creative problem-solving. Experiments targeting both the idea generation and the evaluation phases could manipulate the number of explicit rules participants are provided with and/or the extent in which making associations is necessary to complete the task. Finally, ecologically valid environments could be simulated by providing participants with a vast amount of information and observing how intuition is used to find the relevant clues to the solution.

## Conclusion

Our review showed that intuition is associated with both the idea generation and the idea evaluation phases of the creative problem-solving process. Data was pooled together to obtain a more fine-grained picture about where and how intuitive processes are linked with specific stages of creative problem solving. It was found that previous studies connected intuition chiefly to the idea generation phase. Two possible pathways were sketched out explaining the use of intuition in response to ill-defined problems. Finally, intuition, despite being increasingly investigated in psychological research, is still interpreted in a broad, vague manner, and we suggest future empirical research should be directed to test specific hypotheses such as those offered here or by Sadler-Smith (2015) in order to reveal its underlying working mechanisms in creative problem solving.

## Author Contributions

JP performed the literature review and wrote the manuscript. MO and JB supervised the project and edited the manuscript.

## Conflict of Interest Statement

The authors declare that the research was conducted in the absence of any commercial or financial relationships that could be construed as a potential conflict of interest.
